# The association between healthy eating index-2015 with anthropometric, cardiometabolic and hepatic indices among patients with non-alcoholic fatty liver disease

**DOI:** 10.1186/s12876-024-03222-x

**Published:** 2024-05-10

**Authors:** Seyed Ahmad Hosseini, Ali Akbar Shayesteh, Seyed Jalal Hashemi, Zahra Rahimi, Nader Saki, Hossein Bavi Behbahani, Bahman Cheraghian, Meysam Alipour

**Affiliations:** 1 Nutrition and Metabolic Disease Research Center, Clinical Sciences Research Institute, Ahvaz Jundishapur University of Medical Sciences, Ahvaz, Iran; 2 Alimentary Tract Research Center, Clinical Sciences Research Institute, Ahvaz Jundishapur University of Medical Sciences, Ahvaz, Iran; 3 Hearing Research Center, Clinical Sciences Research Institute, Department of Biostatistics and Epidemiology, School of Public Health, Ahvaz Jundishapur University of Medical Sciences, Ahvaz, Iran; 4 Hearing Research Center, Clinical Sciences Research Institute, Department of Otolaryngology, Head and Neck Surgery, Ahvaz Jundishapur University of Medical Sciences, Ahvaz, Iran; 5 Student Research Committee, Ahvaz Jundishapur University of Medical Sciences, Ahvaz, Iran; 6 Department of Nutrition, Shoushtar Faculty of Medical Sciences, Shoushtar, Iran

**Keywords:** Nonalcoholic fatty liver disease, Healthy eating index, Hepatic steatosis index

## Abstract

**Background:**

Obesity, cardiovascular diseases, and metabolic disorders are common problems among participants with non-alcoholic fatty liver disease (NAFLD). However, the association between these problems and the healthy eating index-2015 (HEI-2015) remains unknown. Although the HEI-2015 originated from American dietary guidelines, its comprehensive evaluation of diet quality provides valuable insights for various populations, including Iranians. Therefore, the objective of this study was to investigate the association between anthropometric, hepatic, and cardio-metabolic indices with HEI-2015 scores in participants with NAFLD.

**Methods:**

We conducted a cross-sectional analysis of data from the Hoveyzeh Cohort Study, which included adults aged 35 to 70 years between 2016 and 2018. A total of 664 participant with NAFLD (452 females and 212 males) were included in the analysis. The HEI-2015 was assessed using the Food Frequency Questionnaire (FFQ). Various indices, including the body shape index (ABSI), atherogenic index of plasma (AIP), visceral adiposity index (VAI), lipid accumulation product (LAP), cardiometabolic index (CMI), lipoprotein combine index (LCI), AST/ALT ratio, ALD/NAFLD index, and hepatic steatosis index (HSI), were calculated.

**Results:**

No significant differences were observed in anthropometric, cardio-metabolic, and hepatic indices across the quartiles of HEI-2015. However, among participants with NAFLD, men had significantly higher AIP and LCI levels, while women had significantly higher BMI, ABSI, VAI, LAP, and CMI levels. Additionally, women with NAFLD exhibited higher AST/ALT and HSI levels but lower ALD/NAFLD levels compared to men with NAFLD. Linear regression analysis among men with NAFLD revealed a significant negative correlation between HEI-2015 score and HSI in both the unadjusted model (β=-0.131, SE = 0.058, *p* = 0.024) and the adjusted model for energy intake (β=-0.129, SE = 0.058, *p* = 0.028).

**Conclusion:**

The present study demonstrated a correlation between lower HEI-2015 scores and an increased risk of steatosis in men with NAFLD. Moreover, our findings highlighted gender-related differences in NAFLD and cardio-metabolic disorders.

## Background

 Nonalcoholic fatty liver disease (NAFLD) is the most prevalent liver condition worldwide [1]. It encompasses a range of hepatic diseases that are associated with comorbidities such as dyslipidemia, hypertension, obesity, and type 2 diabetes mellitus [2]. The global prevalence of NAFLD is estimated to be approximately 25% [3]. The highest prevalence rates have been reported in South America and the Middle East (31% and 32% respectively), followed by Asia (27%), North America (24%), Europe (23%), and Africa (27%), according to studies [4]. The pathophysiology of NAFLD is multifactorial, involving various Factors such as genetic factors, insulin resistance, obesity, and dietary factors [5, 6]. The studies have demonstrated that obesity, particularly abdominal obesity, independently contributes to an increased risk of NAFLD [7]. NAFLD closely interacts with adipose tissue, which functions as an endocrine organ by secreting adipokines [8]. Adipokines are involved in several pathological conditions, including subclinical systemic inflammation, insulin resistance, dyslipidemia, and NAFLD [9]. Furthermore, NAFLD is strongly associated with metabolic syndrome, with approximately 91% of NAFLD participants presenting with at least one component of metabolic syndrome and about 57% have three or more criteria [10]. Additionally, NAFLD has been identified as an independent risk factor for cardiovascular disease and subclinical atherosclerosis [11]. Studies have reported higher 10-year cardiovascular risk scores in individuals with NAFLD compared to healthy subjects [12, 13].

Diet may play a mediating role in the relationship between NAFLD and cardiovascular and metabolic complications. The consumption of energy-dense, nutrient-poor snacks, cakes, biscuits, and soft drinks has been found to increase the risk of NAFLD [14]. Additionally, the role of specific macronutrients such as sugars and saturated fatty acids, as well as the Western dietary pattern characterized by highly processed foods, candies, sweets, sugar-sweetened beverages, refined grains, red meat, and high-fat dairy products, has been identified as crucial in the onset and progression of NAFLD [15]. Conversely, studies have shown that the consumption of omega-3 fatty acids, nuts, green coffee bean extract, dietary antioxidants, and adherence to Mediterranean dietary patterns can help prevent the progression of NAFLD [16–20].

Recent research suggests that assessing diet-disease associations using dietary quality indices is more informative than focusing on single nutrients or food items [21]. The Healthy Eating Index (HEI) was developed by the United States Department of Agriculture (USDA) as a tool to evaluate diet quality based on adherence to the 2015–2020 Dietary Guidelines for Americans [22]. Previous studies have indicated a correlation between lower HEI scores and an increased risk of various diseases [23–25]. Moreover, studies on the Iranian population have indicated a significant correlation between HEI and a reduced risk of NAFLD [26, 27]. However, the association between HEI scores and cardio-metabolic complications related to NAFLD in the Iranian population remains unknown. Therefore, the objective of this study was to investigate the relationship between obesity, hepatic and cardio-metabolic indices (as non-invasive markers), and HEI-2015 scores in participants with NAFLD.

## Methods

### Participants

**Fig. 1:**
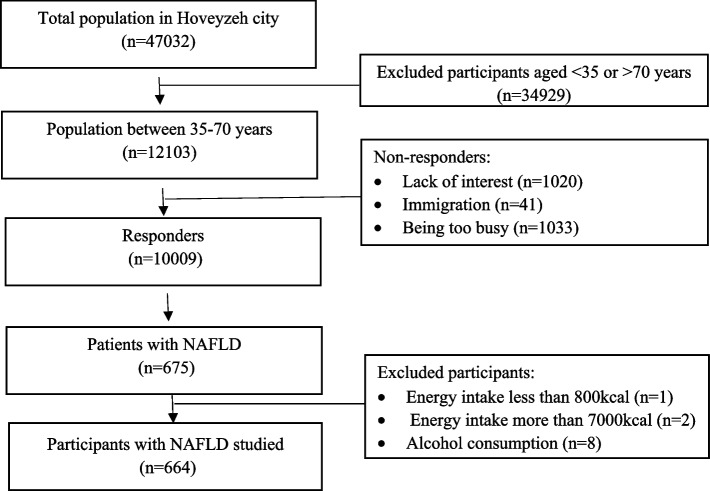
Flow diagram of study selection

The present cross-sectional study was conducted as part of the Hoveyzeh Cohort Study, a prospective population-based study focusing on non-communicable diseases in an Arab community in Southwest Iran [28]. The study included adults aged 35–70 years and was carried out between May 2016 and August 2018. Figure 1 illustrates that out of the 10,009 responders in Hoveyzeh city, a total of 675 participants with NAFLD were evaluated. The inclusion criteria for the study were the presence of NAFLD, willingness to participate, and age range of 35 to 70 years. We excluded 11 participants with NAFLD who did not meet the exclusion criteria: one with an energy intake of less than 800 kcal, two with an energy intake of more than 7000 kcal [29], and eight with alcohol consumption exceeding 2 g per day. Ultimately, the analysis was conducted on 664 participants with NAFLD, consisting of 452 females and 212 males.

### Anthropometric assessment

Body weight was measured using a standing scale (Seca 755) in kilograms (kg), and height was measured using a stadiometer (Seca 206) in centimeters (cm). Waist circumference (WC), wrist circumference, and hip circumference (HC) were measured using Seca locked tape meters, also in centimeters (cm). The body mass index (BMI) was calculated by dividing the body weight (kg) by the square of height (meters, m). The Visceral Adiposity Index (VAI) and A Body Shape Index (ABSI) were calculated using the following formulas: [30, 31]

$$\text{A}\ \text{body}\ \text{shape}\ \text{index} \left(\text{ABSI}\right)=\frac{\text{WC}}{\text{BMI} 2/3\text{*heigh}1/2}$$

Visceral Adiposity Index (VAI):

$$\mathrm{Men}:\;\lbrack\mathrm{WC}/(39.68\:+\:1.88\;\times\;\mathrm{BMI})\rbrack\;\times\;(\mathrm{TG}\;(\mathrm{mmol}/\mathrm L)/1.03)\;\times\;(1.31/\mathrm{HDL}(\mathrm{mmol}/\mathrm L)$$

$$\mathrm{Women}:\;\lbrack\mathrm{WC}/(36.58\:+\:1.89\;\times\;\mathrm{BMI})\rbrack\;\times\;(\mathrm{TG}/0.81)\;\times\;(1.52/\mathrm{HDL})$$

### HEI-2015 calculation

The dietary intake of participants with NAFLD over the past year was assessed using a Food Frequency Questionnaire (FFQ) consisting of 130 food items. The interviewer administered the FFQ and recorded the selected frequency category (day, week, month, or year) for each food item. The frequency categories were then converted to grams per day to estimate the intake of each food item. Nutrient intakes for all food items were calculated using the food ingredient table from the United States Department of Agriculture.

At the end of the study, three individuals who consumed less than 800 kcal or more than 7000 kcal per day were excluded from the analysis. A detailed description of the scoring criteria for the Healthy Eating Index-2015 (HEI-2015) has been provided elsewhere [32]. The HEI-2015 is based on thirteen components, including total fruits (0–5), whole fruits (0–5), total vegetables (0–5), greens and beans (0–5), whole grains (0–10), dairy (0–10), total protein foods (0–5), seafood and plant proteins (0–5), fatty acids (0–10), refined grains (0–10), sodium (0–10), added sugars (0–10), and saturated fats (0–10). The total HEI-2015 score is calculated by summing the scores of all thirteen components, ranging from 0 to 100.

### Biochemical assessments

After a 12-hour overnight fast, a venous blood sample of 10 ml was collected from all participants. The blood samples were then centrifuged, and the resulting serums were stored at -70 °C until further analysis. Mean Corpuscular Volume (MCV) was measured using a hematology autoanalyzer (Nihon Kohden 6510-k, Japan). Serum levels of glucose, triglycerides (TG), total cholesterol (TC), and high-density lipoprotein cholesterol (HDL-C) were measured using a commercial kit (Pars Azmoon, Tehran, Iran). Serum low-density lipoprotein cholesterol (LDL) was calculated using the Friedewald Eq. [33]. Levels of Aspartate transaminase (AST), alanine aminotransferase (ALT), and Alkaline Phosphatase (ALP) were determined using the method recommended by the International Federation of Clinical Chemistry. All these analyses were performed using commercial kits (Pars Azmon Inc).

The atherogenic index of plasma (AIP), lipid accumulation product (LAP), cardiometabolic index (CMI), lipoprotein combine index (LCI), ALD/NAFLD Index, and hepatic steatosis index were calculated using the following formulas [34–37]:

$$\mathrm{Atherogenic}\;\mathrm{index}\;\mathrm{of}\;\mathrm{plasma}:\;\mathrm{Log}\;(\mathrm{TG}/\;\mathrm{HDL}-\mathrm C)$$

$$\mathrm{The}\;\mathrm{Lipid}\;\mathrm{Accumulation}\;\mathrm{Product}:\;\lbrack\mathrm{WC}-65\rbrack\;\times\;\lbrack\mathrm{TG}\rbrack\;\mathrm{in}\;\mathrm{men},\;\lbrack\mathrm{WC}-58\rbrack\;\times\;\lbrack\mathrm{TG}\rbrack\;\mathrm{in}\;\mathrm{women}$$

$$\mathrm{CMI};\;\mathrm{Cardiometabolic}\;\mathrm{index}:\;\mathrm{TG}/\mathrm{HDL}-\mathrm C\;\times\;(\mathrm{Waist}-\mathrm{to}-\mathrm{height})$$

$$\mathrm{The}\;\mathrm{lipoprotein}\;\mathrm{combine}\;\mathrm{index}:\;\mathrm{TC}\ast\mathrm{TG}\ast\mathrm{LDL}/\mathrm{HDL}-\mathrm C$$

$$\mathrm{ANI}:\;\mathrm{ALD}/\mathrm{NAFLD}\;\mathrm{Index}:\;-58.5\;+\;0.637\;(\mathrm{MCV})\;+\;3.91\;(\mathrm{AST}/\mathrm{ALT})\;-\;0.406\;(\mathrm{BMI})\;+\;6.35\;\mathrm{for}\;\mathrm{male}\;\mathrm{gender}$$

$$\mathrm{Hepatic}\;\mathrm{steatosis}\;\mathrm{index}:\;8\;\mathrm x\;(\mathrm{ALT}/\mathrm{AST}\;\mathrm{ratio})\;+\mathrm{BMI}\;(+2,\;\mathrm{if}\;\mathrm{female};\;+2,\;\mathrm{if}\;\mathrm{diabetes}\;\mathrm{mellitus})$$

To assess the presence of fatty liver, abdominal ultrasound scans were performed on the participants with confirmation from a gastroenterologist who was stationed at the cohort center. The ultrasound evaluations were conducted based on the participant’s medical history and in accordance with established protocols.

### Statistical analysis

The data were analyzed using IBM SPSS Statistics software (Version 24) (IBM SPSS Statistics, Armonk, USA). The normality of variables was assessed using the Shapiro-Wilk test. HEI-2015 scores were divided into quartiles for analysis. ANOVA test for quantitative variables (parametric variables) and Kruskal-Wallis test for quantitative variables (non-parametric variables) were employed to compare continuous variables across quartiles of HEI-2015. The Chi-square test was used to compare categorical variables.

Linear regression analysis was conducted in three models to determine the association between the independent variable, HEI-2015 score, and the dependent variables, including anthropometric, metabolic, and hepatic indices. Model 0 represented linear regression analysis without any adjustment, Model 1 included adjustment for energy intake, and Model 2 involved adjustment for age, energy intake, and wrist circumference.

Binary logistic regression analysis was employed to explore the associations of HEI with anthropometric, metabolic, and hepatic indices. Based on previous studies, we considered BMI, ABSI, VAI, AIP, LAP, CMI, LCI, AST/ALT, ANI and HSI cut-off points to be 30, 0.08, 1.78, 0.11, 38, (0.8 for women, 1.748 for men), 16, 1, -0.66 and 36, respectively [38–43]. Values above these cut-off points were considered as the dependent variable in the study. Odds ratios (ORs) and 95% confidence intervals (95% CIs) were reported for the association of HEI-2015 with metabolic and hepatic indices in both crude and adjusted models. Model 1 controlled for age, gender, and energy intake, while the second model further adjusted for education years, diabetes, smoking, and marital status. A p-value less than 0.05 was considered statistically significant.

## Results

The characteristics of participants with NAFLD across the quartiles of HEI-2015 have been shown in Table 1. There were no significant differences observed in age, gender, smoking status, marital status, education level, diabetes, blood pressure, heart rate, anthropometric indices (weight, height, waist circumference, hip circumference), biochemical factors (fasting blood sugar, triglycerides, cholesterol, HDL, LDL, AST, ALT, ALP), and energy intake between the quartiles of HEI-2015 (*p* > 0.05). However, a significant difference was found in the levels of ALT among the groups (*p* = 0.04).

**Table 1:** Baseline characteristics of 664 participants with non-alcoholic fatty liver in a cross sectional study of Hoveyzeh Cohort Study

Variables	HEI quartiles Total (*N* = 664)	HEI quartiles Q1 (*N* = 166)	HEI quartiles Q2 (*N* = 166)	HEI quartiles Q3 (*N* = 166)	HEI quartiles Q4 (*N* = 166)	*P*-Value
Age (years)	48.00 (12)	47.00 (12)	46.00 (12)	49.00 (13)	48.00 (14)	0.23 ^b^
Gender n (%)						0.37 ^c^
Female	452 (68.07)	115 (69.28)	118 (71.08)	104 (62.65)	115 (69.28)	
Male	212 (31.92)	51 (30.72)	48 (28.92)	62 (37.35)	51 (30.72)	
Marital state n (%)						0.05 ^c^
Single	12 (1.81)	2 (1.20)	7 (4.22)	0 (0)	3 (1.81)	
Married	588 (88.56)	153 (92.17)	148 (89.15)	145 (87.34)	142 (85.54)	
Widow	58 (8.73)	11 (7.23)	10 (6.02)	19 (11.45)	18 (10.84)	
Divorced	6 (0.90)	0 (0)	1 (0.60)	2 (1.21)	3 (1.81)	
Current smoking, n (%)						0.20 ^c^
No	560 (84.34)	135 (81.33)	146 (87.95)	135 (81.33)	144 (86.75)	
Yes	104 (15.66)	31 (18.67)	20 (12.05)	31 (18.67)	22 (13.25)	
Education (years)	4.00 (8)	3.00 (7)	4.00 (8)	3.00 (9)	4.50 (11)	0.45 ^b^
Weight (kg)	86.00 (20)	85.75 (21)	83.25 (19)	87.75 (18)	85.75 (20)	0.22 ^b^
Height (cm)	162.85 (12)	161.45 (12)	162.30 (11)	163.50 (14)	162.55 (12)	0.10 ^b^
WC (cm)	107.00 (14)	107.00 (15)	105.25 (14)	108.00 (14)	108.00 (14)	0.08 ^b^
HC (cm)	108.00 (13)	108.00 (15)	107.00 (15)	109.00 (11)	109.00 (12)	0.58 ^b^
Wrist circumference (cm)	18.00 (2)	18.00 (2)	18.00 (2)	18.00 (2)	17.00 (2)	0.11 ^b^
Fasting blood sugar (mg/dl)	101.00 (34)	102.50 (25)	103.00 (44)	101.00 (37)	101.00 (31)	0.78 ^b^
TG (mg/dl)	156.00 (100)	165.00 (120)	154.50 (93)	152.00 (90)	156.00 (101)	0.84 ^b^
Cholesterol (mg/dl)	189.00 (50)	194.00 (54)	182.00 (43)	191.00 (54)	182.50 (54)	0.13 ^b^
HDL (mg/dl)	48.00 (16)	48.50 (16)	47.00 (15)	48.00 (16)	47.50 (16)	0.56 ^b^
LDL (mg/dl)	104.00 (43.35)	106.20 (44.95)	102.00 (37.65)	105.40 (42.60)	105.20 (53.85)	0.36 ^a^
AST (U/L)	17.00 (9)	18.00 (10)	17.00 (10)	18.00 (9)	16.00 (8)	0.29 ^b^
ALT (U/L)	20.00 (17)	20.00 (17)	19.00 (15)	22.00 (16)	19.00 (17)	0.04 ^b^
ALP	210.00 (78)	206.00 (67)	211.50 (91)	214.00 (78)	206.00 (76)	0.50 ^b^
Systolic blood pressure	112.00 (22)	111.00 (21)	110.00 (20)	112.50 (20)	115.00 (23)	0.26 ^b^
Diastolic blood pressure	70.00 (15)	70.00 (12)	70.00 (14)	70.00 (15)	70.00 (15)	0.69 ^b^
Heart rate	78.00 (11)	79.00 (13)	78.00 (12)	77.00 (12)	79.00 (13)	0.18 ^b^
Diabetes, n (%)	209 (31.5)	45 (31.3)	60 (36.1)	52 (31.3)	52 (31.3)	0.37 ^c^
Energy (Kcal)	2894.37 (1203.82)	2944.65 (1382.81)	2960.18 (1205.36)	2858.38 (1053.19)	2847.38 (1314.96)	0.386 ^b^

Data are median (IQR) for quantitative variables and frequency (percent) for qualitative variables

^a^ ANOVA test for quantitative variables (parametric variables)

^b^ Kruskal-Wallis test for quantitative variables (non-parametric variables)

^c^ chi-square for qualitative variables

The mean ± SD values of HEI-2015 and its components are presented in Table 2. The differences in the quartiles of HEI-2015 and its components were statistically significant (*p* < 0.05).

**Table 2:** A comparison between the quartiles of HEI-2015 and its components in participants with non-alcoholic fatty liver

Variables	HEI quartiles Total (*N* = 664)	HEI quartiles Q1 (*N* = 166)	HEI quartiles Q2 (*N* = 166)	HEI quartiles Q3 (*N* = 166)	HEI quartiles Q4 (*N* = 166)	*P*-Value ^a^
Adequacy components						
Total fruits (5)	4.47 ± 0.98	4.12 ± 1.27	4.49 ± 0.93	4.63 ± 0.76	4.64 ± 0.76	< 0.001
Whole fruits (5)	4.88 ± 0.54	4.72 ± 0.86	4.90 ± 0.41	4.96 ± 0.28	4.95 ± 0.40	< 0.001
Total vegetables (5)	4.85 ± 0.48	4.75 ± 0.63	4.84 ± 0.52	4.93 ± 0.27	4.89 ± 0.39	0.003
Greens and beans (5)	4.78 ± 0.69	4.50 ± 1.07	4.79 ± 0.61	4.92 ± 0.32	4.91 ± 0.45	< 0.001
Whole grains (10)	0.55 ± 1.43	0.27 ± 0.43	0.32 ± 0.59	0.43 ± 0.88	1.18 ± 2.53	< 0.001
Dairy (10)	3.56 ± 2.13	3.19 ± 1.98	3.86 ± 2.43	3.66 ± 2.02	3.53 ± 2.04	0.033
Total protein foods (5)	3.06 ± 1.05	2.60 ± 1.04	2.86 ± 0.94	3.23 ± 0.90	3.58 ± 1.04	< 0.001
Seafood and plant proteins (5)	4.43 ± 0.94	3.94 ± 1.21	4.32 ± 0.95	4.69 ± 0.64	4.76 ± 0.58	< 0.001
Fatty acids (10)	3.76 ± 3.37	1.50 ± 2.48	3.52 ± 2.98	4.27 ± 3.22	5.75 ± 3.29	< 0.001
Moderation components						
Refined grains (10)	0.18 ± 1.26	0.16 ± 0.20	0.91 ± 0.76	0.02 ± 0.18	0.61 ± 2.34	< 0.001
Sodium (10)	2.31 ± 2.78	1.41 ± 2.10	1.51 ± 2.28	2.27 ± 2.53	4.06 ± 3.24	< 0.001
Added sugars (10)	4.17 ± 2.62	4.03 ± 2.79	3.473 ± 2.66	4.637 ± 2.45	4.56 ± 2.53	0.022
Saturated fats (10)	9.05 ± 2.22	7.24 ± 3.40	9.47 ± 1.51	9.69 ± 0.95	9.81 ± 0.74	< 0.001
Total score	50.12 ± 6.06	42.33 ± 4.05	48.76 ± 1.09	52.12 ± 0.94	57.27 ± 3.30	< 0.001

The data are presented as mean ± SD.

^a^ Anova was used for comparison of quartiles

The comparison of anthropometric and metabolic indices based on quartiles of HEI-2015 is presented in Table 3. The results indicate that there were no significant differences in anthropometric indices (BMI, ABSI, VAI) and metabolic indices (AIP, LAP, CMI, LCI) according to quartiles of HEI-2015 (*p* > 0.05). Additionally, there were no significant differences in hepatic indices (AST/ALT, ALD/NAFLD index, hepatic steatosis index) based on quartiles of HEI-2015 (*p* > 0.05) (Table 4).

**Table 3:** A comparison between the anthropometric and cardiometabolic indices based on quartiles of HEI-2015

Variables	HEI quartile Total (*N*=664)	HEI quartiles Q1 (*N*=166)	HEI quartiles Q2 (*N*=166)	HEI quartiles Q3 (*N*=166)	HEI quartiles Q4 (*N*=166)	***P***-Value^**1**^
**Body Mass Index**						
Male	30.62 ± 4.70	30.54 ± 5.68	29.20 ± 3.89	30.93 ± 4.12	31.67 ± 4.96	0.06
Female	33.15 ± 5.46	33.40 ± 5.67	32.54 ± 3.38	34.00 ± 5.31	32.76 ± 5.41	0.18
Total	32.34 ± 5.36	32.55 ± 5.81	31.57 ± 5.21	32.86 ± 5.20	32.43 ± 5.15	0.16
*P*-value ^2^	<0.001	0.003	<0.001	<0.001	0.21	
**A Body Shape Index**						
Male	8.13×10^-2^ ± 0.005	8.12×10^-2^ ± 0.003	8.11×10^-2^ ± 0.003	8.11×10^-2^ ± 0.004	8.19×10^-2^ ± 0.004	0.68
Female	8.42×10^-2^ ± 0.005	8.40×10^-2^ ± 0.005	8.43×10^-2^ ± 0.005	8.40×10^-2^ ± 0.005	8.46×10^-2^ ± 0.005	0.80
Total	8.33×10^-2^ ± 0.005	8.32×10^-2^ ± 0.005	8.33×10^-2^ ± 0.005	8.29×10^-2^ ± 0.005	8.37×10^-2^ ± 0.005	0.46
*P*-value ^2^	<0.001	0.001	<0.001	<0.001	0.003	
**Visceral Adiposity Index**						
Male	3.14 ± 2.90	3.48 ± 3.41	3.28 ± 3.50	2.84 ± 2.17	3.06 ± 2.54	0.68
Female	3.44 ± 2.62	3.48 ± 2.65	3.58 ± 2.23	3.20 ± 2.83	3.51 ± 2.52	0.72
Total	3.35 ± 2.71	3.48 ± 2.89	3.49 ± 2.30	3.06 ± 1.96	3.37 ± 2.53	0.44
*P*-value ^2^	0.184	0.99	0.607	0.26	0.29	
**Atherogenic Index of Plasma**						
Male	0.26 ± 0.27	0.31 ± 0.26	0.26 ± 0.29	0.24 ± 0.24	0.25 ± 0.28	0.57
Female	0.14 ± 0.26	0.14 ± 0.27	0.13 ± 0.28	0.13 ± 0.23	0.15 ± 0.26	0.98
Total	0.18 ± 0.27	0.19 ± 0.28	0.17 ± 0.29	0.17 ± 0.24	0.18 ± 0.27	0.90
*P*-value ^2^	<0.001	<0.001	0.009	0.003	0.002	
**Lipid Accumulation Product**						
Male	91.28 ± 61.76	98.33 ± 66.66	85.61 ± 62.62	85.26 ± 47.41	96.90 ± 71.18	0.60
Female	103.28 ± 68.04	107.23 ± 70.66	100.19 ± 83.26	100.74 ± 48.13	104.82 ± 64.06	0.84
Total	99.45 ± 66.32	104.50 ± 69.37	95.97 ± 77.95	94.96 ± 48.31	102.39 ± 66.22	0.47
*P*-value ^2^	0.17	0.24	0.29	0.52	0.93	
**Cardiometabolic index**						
Male	1.50 ± 0.86	1.63 ± 0.82	1.50 ± 1.09	1.38 ± 0.57	1.51 ± 0.95	0.50
Female	1.78 ± 1.16	1.91 ± 1.35	1.71 ± 1.43	1.72 ± 0.73	1.79 ± 0.93	0.53
Total	1.69 ± 1.08	1.83 ± 1.21	1.65 ± 1.34	1.59 ± 0.69	1.70 ± 0.94	0.24
*P*-value ^2^	0.001	0.17	0.37	0.002	0.07	
**Lipoprotein Combine Index**						
Male	27.56 ± 25.54	32.68 ± 28.06	27.47 ± 33.20	24.41 ± 15.94	26.33 ± 24.05	0.38
Female	24.58 ± 26.31	27.08 ± 28.46	22.55 ± 24.38	22.67 ± 17.06	25.88 ± 32.23	0.46
Total	25.53 ± 26.08	28.80 ± 28.37	23.97 ± 27.21	23.32 ± 16.62	26.02 ± 29.89	0.22
*P*-value ^2^	0.03	0.45	0.28	0.04	0.48	

The data are presented as mean ± SD.

T test and Kruskal-Wallis used to comparison between genders

P-V1: Anova was used to comparison of variables based on quartiles of HEI-2015.

P-V2: t-test was used to comparison of total HEI between men and female

**Table 4:** A comparison between the hepatic indices based on quartiles of HEI-2015

Variables	HEI quartiles Total (*N*=664)	HEI quartiles Q1 (*N*=166)	HEI quartiles Q2 (*N*=166)	HEI quartiles Q3 (*N*=166)	HEI quartiles Q4 (*N*=166)	***P***-Value ^**1**^
**AST/ALT**						
Male	0.73 ± 0.24	0.77 ± 0.31	0.68 ± 0.23	0.73 ± 0.23	0.74 ± 0.19	0.39
Female	0.99 ± 0.37	0.98 ± 0.35	1.02 ± 0.34	0.97 ± 0.31	1.01 ± 0.47	0.72
Total	0.91 ± 0.36	0.92 ± 0.35	0.92 ± 0.34	0.88 ± 0.31	0.93 ± 0.43	0.63
*P*-value ^2^	<0.001	<0.001	<0.001	<0.001	<0.001	
**ALD/NAFLD Index**						
Male	-7.71 ± 4.70	-7.21 ± 4.30	-6.94 ± 4.51	-8.15 ± 5.50	-8.40 ± 4.18	0.32
Female	-15.07 ± 5.04	-15.50 ± 5.13	-14.48 ± 5.09	-15.17 ± 4.84	-15.14 ± 5.07	0.48
Total	-12.72 ± 6.01	-12.95 ± 6.21	-12.30 ± 5.99	-12.55 ± 6.11	-13.06 ± 5.73	0.58
*P*-value ^2^	<0.001	<0.001	<0.001	<0.001	<0.001	
**Hepatic Steatosis Index**						
Male	42.93 ± 6.17	42.67 ± 7.87	42.41 ± 5.64	43.11 ± 5.39	43.49 ± 5.73	0.82
Female	44.25 ± 6.06	44.52 ± 6.49	43.39 ± 5.71	45.40 ± 5.87	43.81 ± 6.01	0.07
Total	43.83 ± 6.12	43.95 ± 6.97	43.11 ± 5.69	44.55 ± 5.79	43.72 ± 5.91	0.19
*P*-value ^2^	0.01	0.113	0.317	0.013	0.75	

The data are presented as mean ± SD.

T test and Kruskal-Wallis used to comparison between genders

P-V1: Anova was used to comparison of variables based on quartiles of HEI-2015.

P-V2: t-test was used to comparison of total HEI between men and female

The anthropometric indices levels including BMI (32.15 ± 5.46 vs. 30.62 ± 4.70, *p* < 0.001), ABSI (8.42 × 10 − 2 ± 0.005 vs. 8.13 × 10 − 2 ± 0.005, *p* < 0.001) and VAI (3.44 ± 2.62 vs. 3.14 ± 2.90, *p* = 0.184) were higher in women than men with NAFLD (Table 3).

The levels of metabolic indices, including AIP (0.26 ± 0.27 vs. 0.14 ± 0.26, *p* < 0.001) and LCI (27.56 ± 25.54 vs. 24.58 ± 26.31, *p* = 0.03), were significantly higher in men compared to women with NAFLD. On the other hand, women with NAFLD had higher levels of BMI (33.15 ± 5.46 vs. 30.62 ± 4.70), ABSI (8.13 × 10 − 2 ± 0.005 vs. 8.42 × 10 − 2 ± 0.005), VAI (3.44 ± 2.62 vs. 3.14 ± 2.90), LAP (103.28 ± 68.04 vs. 91.28 ± 61.76), and CMI (1.78 ± 1.16 vs. 1.50 ± 0.86) compared to men with NAFLD (Table 3). Furthermore, the comparison of hepatic indices revealed that women with NAFLD had higher AST/ALT (0.99 ± 0.37 vs. 0.73 ± 0.24, *p* < 0.001) and hepatic steatosis index (44.25 ± 6.06 vs. 42.93 ± 6.17, *p* = 0.01) levels, and lower ALD/NAFLD (-15.07 ± 5.04 vs. -7.71 ± 4.70, *p* < 0.001) index level compared to men with NAFLD (Table 4).

The comparison of anthropometric, metabolic, and hepatic indices based on quartiles of HEI-2015 is presented in Table 5. The linear regression analysis conducted among men with NAFLD revealed a significant negative correlation between HEI-2015 score and hepatic steatosis index in both the unadjusted model (B = -0.131, SE = 0.058, *p* = 0.024) and the adjusted model for energy intake (B = -0.129, SE = 0.058, *p* = 0.028) (Table 5). Additionally, BMI in men showed a positive correlation with HEI-2015 score in both the unadjusted model (B = 0.131, SE = 0.058, *p* = 0.028) and the adjusted model for energy intake (B = 0.142, SE = 0.058, *p* = 0.015). However, in terms of the association between HEI-2015 score as an independent variable and anthropometric, metabolic, and hepatic indices as dependent variables, there was no significant association observed in the unadjusted and adjusted models for other indices (*p* > 0.05) (Table 5).

**Table 5:** The Association between HEI-2015 score with anthropometric and metabolic indices in participants with non-alcoholic fatty liver

Variables	Model 0			Model 1			Model 2		
	β (Unstandardized)	SE	*P*-Value	β (Unstandardized)	SE	*P*-Value	β (Unstandardized)	SE	*P*-Value
**Body mass index**									
Male	0.131	0.058	0.024	0.142	0.058	0.015	0.059	0.047	0.177
Female	-0.018	0.041	0.668	-0.018	0.041	0.661	0.015	0.032	0.650
Total	0.022	0.034	0.513	0.022	0.034	0.551	0.019	0.028	0.489
**A Body Shape Index**									
Male	3.18	10.49	0.762	3.18	10.49	0.762	5.10	0.001	0.190
Female	6.89	5.87	0.241	6.96	5.88	0.238	-9.83	0.001	0.763
Total	6.12	4.863	0.208	5.68	4.93	0.250	1.31	0.001	0.616
**Visceral Adiposity Index**									
Male	-0.008	0.036	0.820	-0.002	0.036	0.947	-0.005	0.035	0.885
Female	-0.011	0.020	0.579	-0.011	0.020	0.573	-0.013	0.020	0.496
Total	-0.010	0.017	0.559	-0.010	0.017	0.582	-0.008	0.017	0.641
**Atherogenic Index of Plasma**									
Male	-0.002	0.003	0.540	-0.002	0.003	0.611	-0.003	0.003	0.400
Female	0.001	0.002	0.895	0.001	0.002	0.902	0.001	0.002	0.929
Total	0.001	0.002	0.833	0.001	0.002	0.910	3.855	0.002	0.998
**Lipid Accumulation Product**									
Male	0.697	0.794	0.362	0.932	0.760	0.221	0.531	0.711	0.456
Female	-0.286	0.510	0.575	-0.291	0.510	0.569	-0.353	0.500	0.480
Total	-0.022	0.425	0.959	-0.004	0.425	0.993	-0.032	0.411	0.938
**Cardiometabolic index**									
Male	0.002	0.011	0.854	0.005	0.011	0.650	0.003	0.011	0.779
Female	-0.011	0.009	0.217	-0.011	0.009	0.216	-0.014	0.009	0.116
Total	-0.007	-0.041	0.292	-0.007	0.007	0.298	-0.008	0.007	0.243
**Lipoprotein Combine Index**									
Male	-0.146	0.316	0.645	-0.127	0.319	0.691	-0.191	0.316	0.547
Female	-0.159	0.197	0.420	-0.160	0.197	0.418	-0.228	0.198	0.250
Total	-0.156	0.167	0.352	-0.150	0.167	0.371	-0.161	0.167	0.337
**AST/ALT**									
Male	-0.003	0.003	0.360	-0.004	0.003	0.234	-0.003	0.003	0.379
Female	0.001	0.003	0.744	0.001	0.003	0.741	0.001	0.003	0.792
Total	-5.50	0.002	0.981	0.002	0.006	0.879	-0.001	0.002	0.790
**Hepatic Steatosis Index**									
Male	-0.131	0.058	0.024	-0.129	0.058	0.028	0.028	0.063	0.662
Female	-0.003	0.038	0.935	-0.003	0.038	0.943	0.006	0.037	0.865
Total	0.008	0.039	0.840	0.010	0.039	0.803	0.011	0.033	0.734
**ALD/NAFLD Index**									
Male	0.095	0.076	0.214	0.119	0.076	0.117	-0.100	0.057	0.081
Female	-0.024	0.035	0.596	-0.025	0.045	0.588	-0.023	0.036	0.523
Total	-0.038	0.038	0.325	-0.033	0.038	0.386	-0.035	0.038	0.355

Model 0: linear regression analysis without adjustment

Model 1: linear regression analysis with adjustment for energy intake

Model 2: linear regression analysis with correction for age, energy intake, wrist circumference and diabetes

The multivariable-adjusted odds ratios (ORs) and 95% confidence intervals (CIs) for HEI scores and cardio-metabolic and hepatic indices are presented in Table 6. The results showed no significant association between HEI score and the odds of cardio-metabolic and hepatic indices. However, after adjusting for age, gender, energy intake, education years, diabetes, smoking, and marital status, a significant positive association was found between HEI scores and BMI (OR: 1.198, 95% CIs: 1.031–1.392, *p* = 0.018) (Table 6).

**Table 6:** Odds ratios between healthy eating index and cardio-metabolic and hepatic indices in a cross sectional study of Hoveyzeh Cohort Study

Variables	Healthy eating index		
	Odd ratio	CI (95%)	***P***-value
**Body mass index**			
Model 1	1.148	0.995-1.324	0.059
Model 2	1.198	1.031-1.392	0.018
**A Body Shape Index**			
Model 1	1.065	0.908-1.249	0.440
Model 2	1.077	0.900-1.287	0.418
**Visceral Adiposity Index**			
Model 1	1.040	0.888-1.219	0.628
Model 2	1.077	0.915-1.268	0.372
**Atherogenic Index of Plasma**			
Model 1	0.935	0.815-10.74	0.343
Model 2	0.925	0.800-1.069	0.291
**Lipid Accumulation Product**			
Model 1	1.106	0.857-1.426	0.439
Model 2	1.128	0.869-1.465	0.365
**Cardiometabolic index**			
Model 1	0.926	0.753-1.139	0.465
Model 2	0.949	0.770-1.170	0.626
**Lipoprotein Combine Index**			
Model 1	1.038	0.904-1.191	0.598
Model 2	1.033	0.898-1.189	0.651
**AST/ALT**			
Model 1	1.031	0.884-1.203	0.684
Model 2	1.048	0.890-1.234	0.573
**Hepatic Steatosis Index**			
Model 1	1.091	0.870-1.369	0.448
Model 2	1.097	0.871-1.381	0.432
**ALD/NAFLD Index**			
Model 1	1.320	0.779-2.235	0.302
Model 2	1.270	0.696-2.318	0.435

Model 1: age, gender and energy intake

Model 2: Adjusted for age, gender, energy intake, education years, diabetes, smoking and marital status

## Discussion

In the present study, we investigated the association between HEI-2015 score and anthropometric, cardio-metabolic, and hepatic indices in participants with NAFLD in the Iranian population. Our findings revealed no significant differences in anthropometric, cardio-metabolic, and hepatic indices based on quartiles of HEI-2015. However, among men with NAFLD, we observed a significant inverse correlation between the total score of HEI-2015 and HSI, as well as a positive correlation with BMI.

Consistent with our findings, Yaoo et al. conducted a cohort study and reported that individuals with higher HEI-2015 scores, indicating better adherence to dietary recommendations, had a lower risk of NAFLD [44]. Similarly, Song-Yi et al. performed a nested case-control analysis and found an inverse association between higher HEI-2015 scores and NAFLD risk in a multiethnic population [45]. In a case-control study conducted by Hashemi Kani et al., it was observed that participants diagnosed with non-alcoholic fatty liver disease (NAFLD) had lower dietary quality scores based on the Healthy Eating Index (HEI) compared to their healthy counterparts [46].

The previous studies have consistently demonstrated the association between components of the HEI-2015 and the risk of NAFLD. Recent findings from a 4.2-year follow-up study in participants with NAFLD indicated that higher consumption of vegetables and fruits was associated with a protective effect against NAFLD and its associated metabolic comorbidities [36]. Furthermore, Bahrami et al. reported that a greater intake of legumes (OR = 0.73), lentils (OR = 0.61), and beans (OR = 0.35) was associated with a lower risk of NAFLD [47].

Moreover, a clinical trial demonstrated that a 12-week consumption of whole grains had beneficial effects on hepatic steatosis and the levels of alanine aminotransferase and aspartate aminotransferase in participants with NAFLD [44]. In terms of dairy consumption, a cross-sectional study reported that higher yogurt consumption (≥ 4 times/week) was associated with a lower odds ratio of newly diagnosed NAFLD compared to those who consumed yogurt less than once a week [48].

Furthermore, a clinical trial indicated that dietary intake of saturated fatty acids promotes fatty liver, while consumption of polyunsaturated fatty acids helps prevent liver fat accumulation and reduces hyperlipidemia in overweight subjects [49]. In our study, we observed that the severity of NAFLD, as assessed by hepatic indices such as HSI, ANI, and ALD/NAFLD, was worse in women compared to men. Conversely, cardio-metabolic indices such as AIP and LCI were more unfavorable in males with NAFLD compared to females. The results of studies evaluating the role of gender in the etiology of NAFLD and its cardio-metabolic outcomes are contradictory.

A cross-sectional analysis demonstrated that NAFLD had a greater adverse influence on lipid profiles in men than in women [50]. Another cross-sectional study by Ni et al. suggested that the effect of NAFLD on type 2 diabetes mellitus was more pronounced in males (OR = 2.442) than in females (OR = 1.814) [51]. However, contradicting our findings, a meta-analysis reported a higher prevalence of cardiovascular events in women with NAFLD compared to men [52].

In young people and before the onset of menopause in women, it seems that the risk of cardiovascular disease is more common in men than women, due to the fact that estrogen has a protective role against cardiovascular problems [53].

Since we did not evaluate menopausal status as a potential confounding factor in our study, it is recommended that future research should consider assessing the association between gender and NAFLD while taking menopausal status into account. A similar study considering menopausal status could provide further insights into the gender differences in NAFLD and its relationship with cardiovascular outcomes.

###  Limitations and future directions

A strength of the present study is large sample size, so that all the people of Hoveyzeh city were investigated. The present study had some limitations. First, FFQ to evaluate dietary intake is based on participants’ memories (which can cause both underestimation and overestimation), and it may introduce recall bias. Second, The HEI was developed by the United States Department of Agriculture and is based on dietary guidelines for Americans, so lack of HEI validation in the Iranian population is another limitation of this study.

Third, lack of histology data, confirmation of the data by histology could lead to a better interpretation of the results. Fourth, the absence of a comparator group comprising individuals without NAFLD. Incorporating this group would have enabled a more detailed analysis of the influence that dietary habits might have on the onset of NAFLD, thereby establishing a more distinct comparison between individuals with and without the condition.

Furthermore, in the current study, the limited range of clinical findings in participants with NAFLD may have limited our ability to thoroughly investigate the correlation between diet quality and hepatic indices in a wider range of NAFLD severity. Therefore, in order to have a more thorough understanding of how diet quality affects the advancement of NAFLD, future studies should encompass a broader spectrum of NAFLD stages.

## Conclusions

The present study demonstrated a correlation between less healthy eating index score and increased risk of steatosis in men with NAFLD. Also, our finding indicated there was gender-related differences in NAFLD and cardio-metabolic problems. However, given some limitations in the present stuy, further research is needed to clarify the association between diet quality and cardio-metabolic complications related to NAFLD in the Iranian population .We also recommend studies with focus on gender as a decisive factor in NAFLD-related cardio-metabolic outcomes.

## Data Availability

The datasets used and/or analyzed during the current study available from the corresponding author on reasonable request.

## Abbreviations

NAFLD: Non-alcoholic fatty liver disease
ALD: Alcoholic liver disease
HEI-2015: Healthy eating index-2015
FFQ: Food Frequency Questionnaire
ABSI: A Body Shape Index
AIP: Atherogenic index of plasma
VAI: Visceral adiposity index
LAP: Lipid accumulation product
CMI: Cardiometabolic index
LCI: Lipoprotein combine index
HIS: Hepatic steatosis index
AST: Aspartate aminotransferase
ALT: Alanine transaminase
BMI: Body mass index
MCV: Mean Corpuscular Volume
TG: Triglycerides
TC: Total cholesterol
HDL-C: High-density lipoprotein cholesterol
LDL: Low-density lipoprotein cholesterol
LAP: Lipid accumulation product
CMI: Cardiometabolic index
LCI: Lipoprotein combine index
WC: Waist circumference
ANI: Alcoholic liver disease /nonalcoholic fatty liver disease index
HSI: Hepatic Steatosis Index
